# Prevalence of low back pain in children and adolescents: a meta-analysis

**DOI:** 10.1186/1471-2431-13-14

**Published:** 2013-01-26

**Authors:** Inmaculada Calvo-Muñoz, Antonia Gómez-Conesa, Julio Sánchez-Meca

**Affiliations:** 1 Department Physiotherapy, Faculty of Medicine, Espinardo Campus, University of Murcia, Murcia, 30100, Spain; 2 Department of Physiotherapy, University of Murcia, Murcia, Spain; 3 Department of Basic Psychology and Methodology, University of Murcia, Murcia, Spain

**Keywords:** Low back pain, Children, Adolescents, Prevalence, Meta-analysis

## Abstract

**Background:**

Low back pain (LBP) is common in children and adolescents, and it is becoming a public health concern. In recent years there has been a considerable increase in research studies that examine the prevalence of LBP in this population, but studies exhibit great variability in the prevalence rates reported. The purpose of this research was to examine, by means of a meta-analytic investigation, the prevalence rates of LBP in children and adolescents.

**Methods:**

Studies were located from computerized databases (ISI Web of Knowledge, MedLine, PEDro, IME, LILACS, and CINAHL) and other sources. The search period extended to April 2011. To be included in the meta-analysis, studies had to report a prevalence rate (whether point, period or lifetime prevalence) of LBP in children and/or adolescents (≤ 18 years old). Two independent researchers coded the moderator variables of the studies, and extracted the prevalence rates. Separate meta-analyses were carried out for the different types of prevalence in order to avoid dependence problems. In each meta-analysis, a random-effects model was assumed to carry out the statistical analyses.

**Results:**

A total of 59 articles fulfilled the selection criteria. The mean point prevalence obtained from 10 studies was 0.120 (95% CI: 0.09 and 0.159). The mean period prevalence at 12 months obtained from 13 studies was 0.336 (95% CI: 0.269 and 0.410), whereas the mean period prevalence at one week obtained from six studies was 0.177 (95% CI: 0.124 and 0.247). The mean lifetime prevalence obtained from 30 studies was 0.399 (95% CI: 0.342 and 0.459). Lifetime prevalence exhibited a positive, statistically significant relationship with the mean age of the participants in the samples and with the publication year of the studies.

**Conclusions:**

The most recent studies showed higher prevalence rates than the oldest ones, and studies with a better methodology exhibited higher lifetime prevalence rates than studies that were methodologically poor. Future studies should report more information regarding the definition of LBP and there is a need to improve the methodological quality of studies.

## Background

The term low back pain (LBP) was defined by Andersson [1] as “pain limited to the region between the lower margins of the 12th rib and the gluteal folds”. LBP is the most common type of back pain [2], occurring in about 60–80% of people at some point in their lives [3].

LBP often begins in childhood, and in adolescents the prevalence is similar to that of adults [4,5]. One characteristic of LBP in childhood and adolescence is its high recurrence and the tendency to reappear with greater intensity [6]. Although initially intensity is usually low [7] and it generally lasts for less than a week [7,8], LBP causes limitations in carrying out activities [9], school absenteeism and the reduction or ceasing of physical activity [10].

In recent years there has been a considerable increase in research studies that examine the prevalence of LBP in this population [7,10-16], but studies exhibit great variability in prevalence rates, with estimates ranging from 1.1% [14] to 66% [7].

This variability found in the prevalence estimates may be due to differences among the studies in such factors as the age of the sample, the sample size, the definition of LBP, the LBP recall period, the strategy for extracting data and the methodology used.

The prevalence of a condition is the number of cases in a specified population at a particular time [17]. The prevalence is described in terms such as: point prevalence (the number of persons in a defined population who had a specified disease or condition at a particular point in time, usually the time the survey was carried out), period prevalence (the number of persons who had a specified disease or condition at any time during a specified time interval), and lifetime prevalence (the number of persons who had a specified disease or condition at some point in their life) [18].

According to the literature on the epidemiology of LBP in children and adolescents, the prevalence rates increase with the age of the subjects [11,19-21] and females have higher prevalence rates than males [12,16,22-24]. Epidemiological studies indicate that the point prevalence is less than the period prevalence and, in turn, this is less than the lifetime prevalence [12,25,26].

Several narrative reviews have been published about the prevalence of LBP in children and adolescents [27-29]. However, to our knowledge, a meta-analysis of the LBP prevalence in children and adolescents has not yet been carried out. Thus, the main purpose of this research was to systematically review the prevalence of LBP obtained in studies with samples composed by children and/or adolescents, in order to: (a) obtain estimates of point, period, and lifetime prevalence rates; (b) determine whether the heterogeneity exhibited by the prevalence rates of the studies can be explained by random sampling alone or, on the contrary, the studies present very discrepant prevalence rates, and (c) in the latter case, to search for substantive and methodological characteristics of the studies that can explain this heterogeneity.

Based on the results of previous research, several hypotheses were raised: (a) the mean estimate of point prevalence will be lower than that of period prevalence and, in turn, this will be lower than that of lifetime prevalence; (b) the prevalence rates will increase with the age of the participants in the samples, and (c) females will present higher prevalence rates than males.

## Methods

In order to accomplish our objectives, a systematic review and a meta-analysis were carried out on observational studies that reported prevalence rates of LBP in children and/or adolescents.

### Selection criteria of the studies

To be included in the meta-analysis, the studies had to meet the following criteria: (a) the study must be an observational study (survey research, longitudinal or cross-sectional design) that reports any prevalence rate of low back pain in children and/or adolescents (≤ 18 years old); (b) the sample size had to have at least 50 participants; (c) studies had to be carried out between 1980 and April of 2011, as previous literature searches carried out by our research team evidenced an absence of studies that report LBP prevalence rates for children/adolescents before 1980; (d) studies had to be written in English, French, Italian, Spanish, or Portuguese; (e) studies could be published or unpublished. Cohort, case–control, controlled clinical trials, and randomized clinical trials were excluded, as they report incidence rates instead of prevalence rates.

### Literature search

To locate studies that met the selection criteria, several literature searching strategies were used. Firstly, the following electronic databases were consulted: ISI Web of Knowledge, MedLine, PEDro, IME, LILACS, and CINAHL. The search period extended to April 2011, with the Medical Subject Headings: adolescents, children, childhood, back pain, low back pain, spinal pain, epidemiology, and prevalence. Secondly, several electronic specialized journals were consulted such as, *Spine*, *Spine Journal*, *Pain*, *European Spine Journal*, *Fisioterapia, Scandinavian J Public Health*, *European Journal of Public Health*, and, finally, the reference lists of the studies recovered were also consulted.

Two reviewers independently: (a) screened the title and abstract of each reference to locate potentially relevant studies, and once hardcopies of the screened papers were obtained, (b) reviewed them in detail to identify articles that fulfilled the selection criteria.

**Figure 1:**
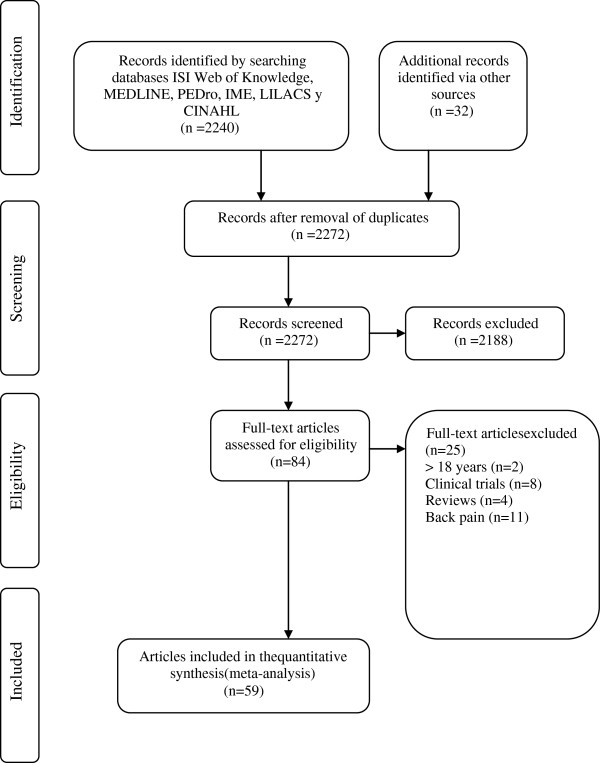
Flow chart of the selection of studies for the meta-analysis.

The total number of references identified with all of the searching strategies used was 2,272, of which 2,188 were excluded in a first screening. The main reasons for deleting these studies were because the participants in the samples were adults (about 55%), the studies did not report prevalence rates (about 20%), the studies were clinical trials (about 15%), or other reasons (about 10%). The searching process enabled us to select 59 articles that met the selection criteria, with a total sample size of 125,483 participants. Figure 1 presents the flow chart of the selection process of the studies.

### Data extraction

With the purpose of identifying characteristics of the studies that may affect the prevalence rates estimated, a codebook was produced that included a series of potential moderator variables of the prevalence rates of LBP. The study characteristics were classified into substantive (subject and context variables), methodological, and extrinsic variables [30].

The following subject characteristics were coded: (a) the origin of the sample (school, community, university, or sport origin); (b) the mean age of the sample (in years), and (c) the gender distribution of the sample (percentage of males). Two context characteristics were coded: the continent and the country where the study was carried out.

The following methodological characteristics were coded: (a) the initial sample size of the study; (b) the final sample size (subjects actually used to calculate the prevalence rate); (c) the response rate (percentage of people in the final sample divided by the number of people in the initial sample); (d) the study design (longitudinal *vs.* cross-sectional); (e) the data extraction method (questionnaire, interview, physical examination); (f) the type of prevalence reported (point, period, or lifetime prevalence); (g) the methodological quality of the study, assessed with an instrument used in previous systematic reviews on the prevalence of LBP [31-33]. This instrument contains 12 items grouped in three clusters focusing on the sample representativeness, the quality of the data collected, and the clarity of the definition of LBP, see Additional file 1. Finally, two extrinsic characteristics were coded: the publication date (year) and the publication source (published vs. unpublished).

To assess the reliability of the coding process, two researchers independently coded 14 studies randomly selected from the total set of studies (24%). The inconsistencies between the two coders were solved by consensus, and when these were due to ambiguity in the codebook, this was corrected. The codebook can be obtained from the corresponding author upon request. To assess the coding reliability, intra-class and kappa coefficients were calculated for the continuous and categorical variables, respectively. On average, the intra-class correlation was 0.994 (range: 0.98 - 1.0), and the kappa coefficient was 0.989 (range: 0.77 - 1.0), all of which can be considered to be highly satisfactory [34].

### Prevalence rates

Three types of prevalence were included in our review: point, period, and lifetime prevalence. In turn, period prevalence was classified according to the specific period of time reported in the study (one and two weeks, 1, 3, 6, and 12 months). From each study, at least one prevalence rate could be obtained. When a study applied a longitudinal design, only the prevalence rate at the beginning of the study was recorded in our meta-analysis. To assess the reliability in the calculations of the prevalence rates, the same 14 studies used to assess the coding reliability of the moderator variables were used. The mean intra-class correlation was 0.999 (range: 0.996 – 1.0), which was highly satisfactory [34].

### Statistical analysis

As each study could report more than one prevalence rate (e.g., point and lifetime prevalences), separate meta-analyses were carried out for the different types of prevalence in order to avoid dependence problems. To normalize the distribution of the prevalence rates, these were transformed by means of the logit event rate: *Lp* = *Lnp*/(1 − *p*)], *p* being the prevalence rate, *Ln* the natural logarithm, and *Lp* the logit event rate. The sampling variance of each logit event rate, *V*(*Lp*), was calculated by means of: *V*(*Lp*) = 1/(*np*) + 1/*n*(1 − *p*)], with *n* being the sample size. Once the statistical analyses were carried out, the results were back-transformed to prevalence rates in order to facilitate their interpretation, by means of: *p* = *e*^*LP*^/(*e*^*LP*^ + 1), with *e* being the base of the natural logarithm [35].

In each meta-analysis, a random-effects model was assumed to calculate a mean prevalence rate and a 95% confidence interval [36]. The random-effects model was assumed because it is the most realistic assumption when the studies to be meta-analyzed can be reasonably considered a representative sample of a population of potential studies that have been conducted or that can be conducted in the future about the topic. The random-effects model allows a higher generalization of the results than the fixed-effects model [37].

The heterogeneity of the prevalence rates was assessed with the *Q* statistic and the *I*^2^ index. When the number of prevalence estimates was large enough (20 or more studies), a forest plot was constructed. In order to test whether publication bias might be a threat to the validity of the prevalence estimates, funnel plots were constructed and Duval and Tweedie’s trim-and-fill method was applied [38].

When the *Q* statistic was statistically significant and the *I*^2^ index exhibited a large value (over 25%), then analyses of potential moderator variables were carried out. However, due to the small number of studies, these analyses were applied only for the lifetime prevalence rate, as this was reported in 30 studies. The analyses of moderator variables were carried out by assuming a mixed-effects model. For the categorical moderator variables, weighted ANOVAs were calculated, with the *Q*_B_ statistic assessing the statistical significance of the moderator variable on the prevalence rates, and the *Q*_W_ statistic assessing the model misspecification. For the continuous moderators, weighted simple meta-regressions were applied, with the *Q*_R_ statistic testing the statistical significance of the moderator variable, and the *Q*_E_ statistic assessing the model misspecification. In all of the moderator analyses, the proportion of variance accounted for by the moderator variable, *R*^2^, was calculated following Raudenbush’s proposal [39]. In particular, $R^{2}=1-{\hat{\tau }}_{\text{Res}}^{2}/{\hat{\tau }}_{\text{Total}}^{2}$, with ${\hat{\tau }}_{\text{Res}}^{2}$ and ${\hat{\tau }}_{\text{Total}}^{2}$ being the estimated residual and total heterogeneity variances, respectively [40]. In order to find the subset of moderator variables that can explain most of the prevalence rates variability, a multiple meta-regression model (by assuming a mixed-effects model) was adjusted. The moderator variables included in the model were selected taking into account the statistical significance achieved in the previous bivariate analyses. This regression model allowed us to identify the most relevant study characteristics to explain the variability exhibited by the prevalence rates.

The statistical analyses were carried out with the meta-analysis program Comprehensive Meta-analysis 2.0 [41] and with the SPSS macros elaborated by D. B. Wilson [42]. The PRISMA checklist was used to check the reporting quality of the meta-analysis (Additional file 2).

## Results

### Descriptive characteristics of the studies

Fifty-nine articles fulfilled the selection criteria [4,8-12,14,19-26,43-86], and were carried out between 1984 and 2010, see Figure 1. The individual characteristics of each of the integrated studies are presented in Additional file 3 (This file contains data collected from other articles [87-103]).

The sample size distribution was very skewed, with a median of 622 subjects and minimum and maximum values of 88 [22] and 34,423 [43], respectively. Most of the studies were carried out in Europe (42 studies; 73%), followed by North America (6 studies; 10.2%), Oceania and Asia with four studies each (6.8%), and Africa with two studies (3.4%). Only three of the 59 studies had not been published. Fifty-five studies were written in English, three in Portuguese, and one in Spanish.

Three subject characteristics were coded: the origin of the sample, the mean age, and the percentage of males in the sample. Most of the samples were recruited from schools (46 studies; 78%), followed by the community (7 studies, 11.9%). On average, the mean age of the samples was 13.6 years old (range: 9–18.4) and the mean percentage of males was 51.1% (range: 41.5% – 100%).

With regards to the methodological variables, the mean response rate was 83.8%, and most of the studies used a cross-sectional design (52 studies, 88.1%). The data extraction method most commonly used was the questionnaire (46 studies, 78%), followed by the questionnaire and physical examination combined (5 studies, 8.5%), the interview (4 studies, 6.8%), the questionnaire and interview combined (2 studies, 3.4%), and the interview and physical examination combined (2 studies, 3.4%).

Out of the different types of prevalence, period prevalence was the most commonly reported (25 studies, 42.4%), followed by the lifetime and period prevalence combined (15 studies, 25.4%), the lifetime prevalence alone (eight studies, 13.5%), the lifetime, period, and point prevalences combined (five studies, 8.5%), the lifetime and point prevalences combined (three studies, 5.1%), the point prevalence alone (two studies, 3.4%), and the period and point prevalences combined (one study, 1.7%).

The methodological quality scores of the included studies are reported in Additional file 4. The minimum and maximum scores were 40% and 100%, respectively, with a mean of 76.7%, and five studies reaching the maximum score of 100%.

For the first block of methodological questions concerning the sample representativeness (questions 1, 2 and 3 of Additional file 1), 74.6% of the studies used at least one of the following conditions: an entire target population, randomly selected sample, or sample stated to represent the target population. With regards to nonresponse, 23.7% of the studies specified at least one of the following situations: reasons for nonresponse described, nonresponders described, comparison of responders and nonresponders, or comparison of sample and target population. Finally, 86.4% of the studies reported the response rate.

In relation to questions related to the quality of the data (questions 4, 5, 6, 7, 8 and 9 of Additional file 1), the majority of the studies reported primary data on LBP (57 studies). LBP data were collected directly from each child or adolescent in 79.7% of the studies, with the remaining studies being collected from a proxy. Out of the 59 studies, 58 used the same mode of data collection for all participants in the sample.

To collect the data, the studies used different instruments, such as questionnaires (79.7%), interviews (8.5%), and examinations (11.9%). The instruments had to be validated or at least its reliability had to be tested.

With regards to the definition of LBP (questions 10, 11 and 12 of Additional file 1), 61% of the studies applied a precise anatomic delineation of the lumbar area or made a reference to an easily obtainable article that contained such specification. A further useful specification of the definition of LBP was applied in 52.5% of the studies by means of questions such as the frequency, duration or intensity, and character of the pain. 96.6% of the studies used recall periods that were clearly stated (1 and 2 weeks, 1, 3, 6 and 12 months or lifetime).

### Mean prevalence and heterogeneity testing

**Figure 2:**
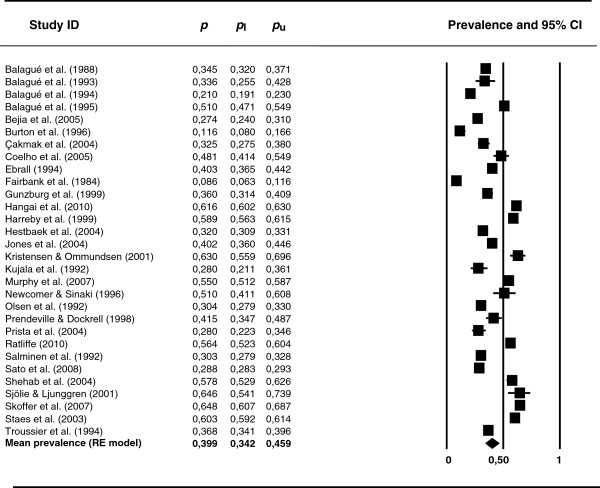
**Forest plot of the lifetime prevalence rates.***p*: prevalence rates. *p*_l_ and *p*_u_: lower and upper confidence limits of the 95% confidence interval around the mean prevalence rate. The last row in the figure presents the mean prevalence assuming a random-effects model.

Table 1 presents the mean rates and 95% confidence intervals for each one of the types of prevalence, as well as the minimum and maximum prevalence rates, and the heterogeneity statistics (*Q* and *I*^2^). The mean point prevalence obtained from 10 studies was 0.120 (95% confidence limits: 0.09 and 0.159). As expected, period and lifetime prevalence rates were higher than point prevalence. Thus, the mean period prevalence at 12 months obtained from 13 studies was 0.336 (95% confidence limits: 0.269 and 0.410), whereas the mean period prevalence at one week obtained from six studies was 0.177 (95% confidence limits: 0.124 and 0.247). The mean lifetime prevalence obtained from 30 studies was 0.399 (95% confidence limits: 0.342 and 0.459). Figure 2 presents a forest plot of the 30 lifetime prevalence rates. The lifetime prevalence estimates exhibited a large heterogeneity, with such extreme minimum and maximum values as 0.086 [52] and 0.648 [9], respectively.

**Table 1:** Mean prevalences, 95% confidence intervals, and heterogeneity statistics

**Type of prevalence**	***k***	***N***	***Min.***	***Max.***	***p***_**+**_	**95%**	**C. I.**	***Q***	***df***	***I***^**2**^
						***p***_**l**_	***p***_**u**_			
Point prevalence	10	49,124	0.032	0.350	0.120	0.090	0.159	546.87*	9	98.3
Period prevalence:										
1 week	6	9,812	0.097	0.350	0.177	0.124	0.247	156.56*	5	96.8
2 weeks	1	1,193	0.246	0.246	0.246	0.222	0.271	--	--	--
1 month	14	23,191	0.025	0.398	0.183	0.128	0.255	1618.85*	13	99.2
3 months	2	4,126	0.224	0.513	0.355	0.134	0.662	187.49*	1	99.5
6 months	7	25,037	0.008	0.420	0.177	0.088	0.324	2197.00*	6	99.7
12 months	13	19,673	0.174	0.603	0.336	0.269	0.410	997.00*	12	98.8
Lifetime prevalence	30	61,732	0.086	0.648	0.399	0.342	0.459	4957.79*	29	99.4

* *p* < .001. *k*: number of studies. *N*: total sample size. *Min.* and *Max.*: minimum and maximum prevalence rates. *p*_+_: mean prevalence. *p*_l_ and *p*_u_: lower and upper confidence limits of the 95% confidence interval around the mean prevalence. *Q*: heterogeneity statistic to test the homogeneity hypothesis of the prevalence rates. *df*: degrees of freedom of the *Q* statistic. *I*^2^: heterogeneity index.

For all of the types of prevalence, the heterogeneity *Q* statistic reached statistical significance (*p* < .001) and all of the *I*^2^ indices were over 95% (see Table 1). Therefore, an analysis of the potential influence of moderator variables was in order. However, the small number of studies that reported point and period prevalence rates discouraged the application of additional analyses on these rates. Therefore, moderator analyses were carried out for the lifetime prevalence rates only.

### Analysis of publication bias

**Figure 3:**
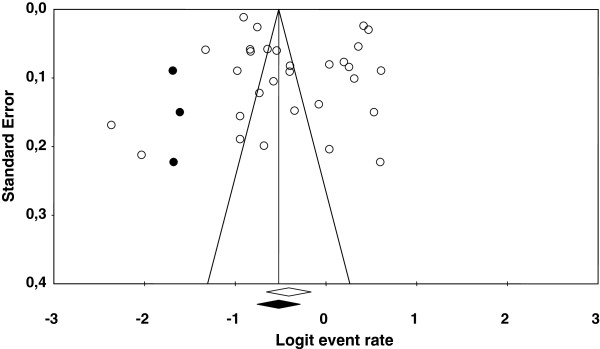
**Funnel plot of the lifetime prevalence logits.** The three full circles are imputed logits by means of the Duval and Tweedie’s trim-and-fill method.

Out of the 30 studies that reported lifetime prevalence estimates, 29 of them were published studies. In order to check whether publication bias might be biasing the estimated mean prevalence, a funnel plot was constructed, as presented in Figure 3. As the figure shows, a slightly higher concentration of prevalence estimates was on the right side of the mean prevalence leading to a slight asymmetry of the funnel plot. By applying Duval and Tweedie’s trim-and-fill method [38], three additional prevalence estimates should be imputed to the set of original prevalence estimates to achieve symmetry in the funnel plot. The mean prevalence rates obtained with the 30 original prevalence rates and after imputing three estimated prevalences to the original ones were very similar: 0.399 and 0.372, respectively. Therefore, we can discard publication bias as a threat to the validity of the estimated mean lifetime prevalence.

Similar analyses were carried out for the other types of prevalence that were represented in the meta-analysis with at least 10 prevalence estimates. This was the case for point prevalence and for one and 12 months period prevalences. Point prevalence exhibited a funnel plot that was very close to symmetry, and only one new prevalence rate had to be imputed to the 10 original prevalence estimates to adjust the funnel plot to symmetry, leading to original (*p*_+_ = 0.120) and adjusted (*p*_+adj_ = 0.103) mean point prevalences that were very similar. With regards to one month period prevalence, the trim-and-fill method did not have to imput any new prevalence rates to achieve symmetry in the funnel plot. On the contrary, the 12 month period prevalence needed to add five new prevalence rates on the left side of the funnel plot to achieve symmetry, leading to a clear decrease in the mean prevalence from 0.336 (with the 13 original prevalence rates) to 0.245 (when imputing five new prevalence rates to the original ones). Therefore, although the point and one month period prevalences do not seem to be affected by publication bias, the 12 month period prevalence showed an overestimation of the population prevalence rate.

### Analyzing moderator variables

**Figure 4:**
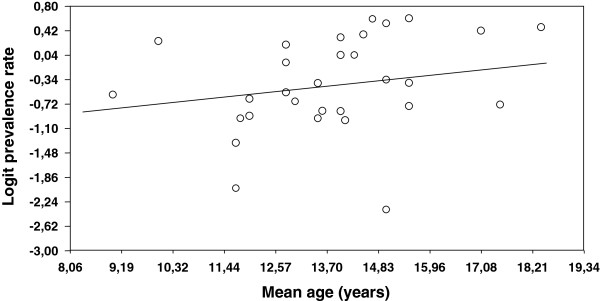
Dispersion diagram of the simple meta-regression of the mean age of the subjects and the logit prevalence rates.

As mentioned above, the analysis of moderator variables was carried out for the lifetime prevalence rates only, as the other types of prevalence were represented in the meta-analysis with a small number of estimates (under 20). Tables 2 and 3 present the results of applying ANOVAs and simple meta-regressions on the subject characteristics coded from the studies. The mean age of the participants in the samples exhibited a positive, statistically significant relationship with the prevalence rates, with a 45.7% of variance accounted for (Table 3). Figure 4 presents a dispersion diagram that illustrates this relationship. Other subject characteristics did not reach a statistically significant relationship with the prevalence rates, such as the origin of the sample (Table 2) and the percentage of males in the sample (Table 3).

**Table 2:** Results of the weighted ANOVAs of qualitative moderator variables on the lifetime prevalence estimates

**Moderator variable**	***k***	***p***_**+**_	**95%**	**C. I.**	**ANOVA results**
			***p***_**l**_	***p***_**u**_	
*Continent*:					
Europe	21	0.390	0.314	0.472	*Q*_B_(4) = 1.95, *p* = .745; *R*^2^ = 0.0
North America	3	0.455	0.255	0.670	*Q*_W_(25) = 4227.25, *p* < .001
Oceania	1	0.403	0.128	0.757	
Africa	2	0.277	0.114	0.532	
Asia	3	0.490	0.285	0.698	
*Origin of the sample*:					
School	25	0.398	0.333	0.467	*Q*_B_(3) = 0.87, *p* = .833; *R*^2^ = 0.0
Community	2	0.396	0.197	0.638	*Q*_W_(26) = 3833.03, *p* < .001
University	2	0.470	0.249	0.703	
School + sport	1	0.280	0.085	0.619	
*Data extraction method:*					
Questionnaire	23	0.411	0.354	0.471	*Q*_B_(1) = 0.36, *p* = .546; *R*^2^ = 0.143
Interview	4	0.365	0.243	0.507	*Q*_W_(25) = 2345.19, *p* < .001

*k*: number of studies. *p*_+_: mean prevalence. *p*_l_ and *p*_u_: lower and upper confidence limits of the 95% confidence interval around the mean prevalence. *Q*_B_: between-categories statistic to test the influence of the moderator variable on the prevalence rates. *Q*_W_: within-categories statistic to test the model misspecification. *R*^2^: proportion of variance accounted for by the moderator variable.

**Table 3:** Results of the weighted meta-regressions of continuous moderator variables on the lifetime prevalence estimates

**Moderator variable**	***k***	***b***_**j**_	***Q***_**R**_	***p***	***Q***_**E**_	***p***	***R***^**2**^
Mean age (years)	30	0.104	5.10	.024	2017.26	< .001	0.457
Gender (% of males)	27	0.008	0.34	.557	4819.51	< .001	0.0
Response rate	27	−0.023	2.94	.086	4807.91	< .001	0.0
Quality scale (0–100)	30	0.019	6.21	.013	2886.94	< .001	0.348
Publication year	30	0.062	9.82	.002	4957.61	< .001	0.0

*k*: number of studies. *b*_j_: regression coefficient of the moderator variable. *Q*_R_: statistic to test the influence of the moderator variable on the prevalence rates. *Q*_E_: statistic to test the model misspecification. *R*^2^: proportion of variance accounted for by the moderator variable.

The continent where the study had been carried out did not affect prevalence rates (Table 2). However the publication year presented a statistically significant relationship with the prevalence rates, with the most recent studies showing higher prevalence rates than the oldest ones (Table 3).

Several methodological variables were also coded in order to examine their potential influence on the prevalence rates. Thus, the data extraction method did not show a statistical relationship with the prevalence rates (Table 2), although the percentage of variance accounted for was not negligible at 14.3%, and the questionnaires (*p*_+_ = 0.411) giving slightly higher prevalence rates than the interviews (*p*_+_ = 0.365). The response rate did not show a statistical relationship with the prevalence rates (Table 3). However, with 34.8% of variance accounted for, the total score of the scale used to assess the methodological quality of the studies exhibited a positive, statistically significant relationship with the prevalence rates, that is, studies with a better methodology tended to show higher lifetime prevalence rates than studies that were methodologically poor (Table 3).

Table 4 presents the results of analyzing the influence of each item of the methodological quality scale on the lifetime prevalence rates. Out of the twelve quality criteria, two of them showed a statistically significant relationship with the prevalence rates. One of them was whether the non response rate was described in the study (*p* = .036, *R*^2^ = 0.676), with higher prevalence rates when the study described the non response. The other one was whether the study clearly defined the specific area of pain (*p* < .001, *R*^2^ = 0.376), with higher prevalence rates in the studies with a clear specification of the area of pain.

**Table 4:** Results of the weighted ANOVAs of quality items on the lifetime prevalence estimates

**Quality item**	***k***	***p***_**+**_	**95%**	**C. I.**	**ANOVA results**
			***p***_**l**_	***p***_**u**_	
*Target population described*:					
Yes	21	0.416	0.346	0.489	*Q*_B_(1) = 0.77, *p* = .380; *R*^2^ = 0.0
No	9	0.358	0.260	0.469	*Q*_W_(28) = 4942.26, *p* < .001
*Non response described*:					
Yes	5	0.486	0.401	0.572	*Q*_B_(1) = 4.42, *p* = .036; *R*^2^ = 0.676
No	20	0.384	0.343	0.427	*Q*_W_(23) = 1162.44, *p* < .001
*Response rate reported*:					
Yes	27	0.403	0.343	0.467	*Q*_B_(1) = 0.21, *p* = .648; *R*^2^ = 0.0
No	3	0.358	0.202	0.551	*Q*_W_(28) = 4910.99, *p* < .001
*Direct reporting from subjects*:					
Yes	26	0.411	0.344	0.480	*Q*_B_(1) = 0.87, *p* = .352; *R*^2^ = 0.0
No	4	0.326	0.190	0.498	*Q*_W_(28) = 4853.39, *p* < .001
*Validated instrument*:					
Yes	26	0.400	0.339	0.464	*Q*_B_(1) = 1.32, *p* = .250; *R*^2^ = 0.015
No	3	0.517	0.332	0.698	*Q*_W_(27) = 4754.11, *p* < .001
*Delimitation of pain*:					
Yes	20	0.472	0.413	0.532	*Q*_B_(1) = 17.98, *p* < .001; *R*^2^ = 0.376
No	10	0.266	0.205	0.338	*Q*_W_(28) = 2192.08, *p* < .001
*Specification of pain*:					
Questionnaire	16	0.399	0.316	0.487	*Q*_B_(1) = 0.00, *p* = .999; *R*^2^ = 0.0
Interview	14	0.399	0.311	0.494	*Q*_W_(28) = 4635.79, *p* < .001

*k*: number of studies. *p*_+_: mean prevalence. *p*_l_ and *p*_u_: lower and upper confidence limits of the 95% confidence interval around the mean prevalence. *Q*_B_: between-categories statistic to test the influence of the moderator variable on the prevalence rates. *Q*_W_: within-categories statistic to test the model misspecification. *R*^2^: proportion of variance accounted for by the moderator variable. Five of the 12 quality items were not included in the table due to one of their categories being represented by one study only or by none (survey designed for studying prevalence rates, data collection was uniform, validated interview, validated exam, and memory period clear).

### An explanatory model

Several substantive and methodological characteristics of the studies showed a statistical relationship with the lifetime prevalence rates, so a multiple regression model can offer a predictive model of the expected prevalence under certain conditions. The predictor variables included in the model were the mean age of the participants in the sample, the publication year, and two methodological variables: the total score on the quality scale (0–100) and the quality item “delimitation of pain” (Yes, 1; No, 0). As shown in Table 5, the predictive model reached a statistically significant relationship with the prevalence rates (*p* < .001, *R*^2^ = 0.438). Two of the four predictive variables showed a statistically significant relationship with the prevalence rates once the other predictors were controlled: the delimitation of pain (*p* = .041) and the publication year (*p* = .013). With the regression coefficients shown in Table 5, it is possible to make predictions about what is the expected lifetime prevalence under certain conditions. The model was however misspecified, so other study characteristics may be influencing the lifetime prevalence rates obtained in the studies.

**Table 5:** Results of the multiple meta-regression on the lifetime prevalence estimates

**Predictor variable**	***b***_**j**_	***Z***	***p***	**Results of the full model**
Constant	−80.160	−2.49	.013	
Delimitation of pain	−0.516	−2.05	.041	*Q*_R_(4) = 27.24, *p* < .001
Mean age (years)	0.029	0.55	.584	*R*^2^ = 0.438
Quality scale (0–100)	0.005	0.63	.531	*Q*_E_(25) = 1306.02, *p* < .001
Publication year	0.040	2.49	.013	

*b*_j_: partial regression coefficient of each moderator variable. *Z*: statistic to test the influence of each moderator variable on the prevalence rates. *Q*_R_: statistic to test the statistical significance of the full model. *Q*_E_: statistic to test the model misspecification. *R*^2^: proportion of variance accounted for by the moderator variables.

## Discussion

The aim of this research was to examine, by means of a meta-analytic investigation, the prevalence rates of LBP in children and adolescents, as well as to search for characteristics of the studies that can explain the heterogeneity exhibited by the prevalence rates. With this purpose, a total of 59 studies fulfilled our selection criteria and were included in our meta-analysis.

The results confirmed the hypothesis that the lifetime prevalence is higher than the period prevalence and, in turn this is higher than the point prevalence. These results coincide with those obtained by Louw et al. [33] that detected mean LBP point, one-year, and lifetime prevalences for adolescents of 12%, 33%, and 36%, respectively.

The hypothesis that the prevalence rate would increase with the age of the participants in the samples was confirmed by our results. These findings are in line with those obtained by Balagué et al. [11] and Jones et al. [19]. Balagué et al. [11] reported lifetime prevalences of 16% and 58% for children and adolescents, respectively, and Jones et al. [19] reported lifetime prevalences of 18.2% for 10 years old children and of 65.6% for 16 year old adolescents.

With regards to gender, authors such as Shebad et al. [20] and Kovacs et al. [62] found higher lifetime prevalences for women (64.7% and 69.3%, respectively) than for men (50.8% and 50.9%, respectively). On the contrary, Newcomer et al. [72] reported higher prevalence rates for men than for women (57% and 44%, respectively). Finally, Olsen et al. [74] did not find relevant differences between the lifetime prevalence rates for men and women (30.7% and 30%, respectively). The results of our meta-analysis exhibited a nonstatistically significant relationship between gender and lifetime prevalence and, as a consequence, our hypothesis of a larger prevalence for women than for men was not confirmed.

The large heterogeneity found in all types of prevalence indicates the existence of characteristics of the studies causing this variability. The publication year influenced the lifetime prevalence rates, with the higher prevalence rates being reported in the most recent studies. This result seems to be very solid, as in the multiple meta-regression model publication year was one of the two predictors that achieved a statistically significant relationship with the lifetime prevalence, once controlled the methodological quality of the studies, the delimitation of pain, and the mean age of the sample. However, what our analyses do not enable us to determine are the reasons of that increase in the prevalence rates. Thus, it could be that children are changing their activity level or they are more overweight; it could be that the actual prevalence has not changed but the reporting has; or it could be that the questions used to assess the prevalence have changed. This point would need more research. Regarding the methodological quality of the studies, the studies with a better quality tended to show higher lifetime prevalence rates than studies that were methodologically poor.

The most common methodological shortcomings of the studies included in the review were the lack of a clear definition and delimitation of LBP and the absence of important specifications of LBP such as the frequency of episodes, its intensity and duration. In particular, out of the studies published between 1980 and 2001, only 40.9% reported a clear definition of LBP, whereas in the most recent studies (from 2002 to date) this percentage was 73%. With regards to the LBP specifications, the percentage was 71% for studies published in the last 10 years and 29% for those that were not published in the last 10 years.

### Limitations of the meta-analysis

It is important to note some limitations of our meta-analysis. The small number of studies that reported point and period prevalence rates discouraged the analysis of moderator variables and, as a consequence, this kind of analysis was applied for lifetime prevalence rates only. On the other hand, the absence of a more detailed description in the primary studies about such important aspects as the exact area of pain caused uncertainty in our coding process.

### Implications for clinical practice

The results of our meta-analysis have important consequences for professionals. Our finding of higher prevalence rates in the most recent studies suggests that LBP is a problem that is increasing in childhood and adolescence. As a consequence, more attention should be devoted to develop and apply prevention programs for young children in order to break this trend. On the other hand, our finding of higher prevalence rates in the studies with older subjects points to the need for efforts towards an early detection of LBP in children and adolescents.

### Implications for future research

Our results enable us to make recommendations for future research in this field. Firstly, it is advisable that future studies report more information regarding the definition of LBP. Issues relating to the exact area of pain, the frequency of episodes, their intensity, and the duration need to be specified. Secondly, there is a need to improve the methodological quality of studies in order to avoid threats against the representativeness of the samples and the internal validity of the studies. Finally, studies should report prevalence rates of LBP disaggregated by age and sex.

## Conclusion

In conclusion, the most recent studies seem to show higher prevalence rates than the oldest ones, and studies with a better methodology tend to show higher lifetime prevalence rates than studies that are methodologically poor.

## Competing interests

The authors declare that they have no competing interests.

## Authors’ contributions

All authors contributed to the conception and design, acquisition, analysis and interpretation of data and drafting of the manuscript. AGC and JSM participated in the critical revision of the manuscript for important intellectual content. ICM and JSM performed the statistical analyses. All authors read and approved the final manuscript.

## Pre-publication history

The pre-publication history for this paper can be accessed here:

http://www.biomedcentral.com/1471-2431/13/14/prepub

## Supplementary Material

Additional file 1 Methodological criteria of epidemiological studies. Click here for file

Additional file 2 PRISMA Checklist. Click here for file

Additional file 3 Characteristics of the studies presenting LBP prevalence data used in this meta-analysis. Click here for file

Additional file 4 Methodological Quality of the 59 epidemiological studies. Click here for file
