# Supplementary material for: Prevalence of low back pain in children and adolescents: a meta-analysis
Source: BMC Pediatr. 2013 Jan 26;13:14. doi: 10.1186/1471-2431-13-14 (PMC3571904; doi:10.1186/1471-2431-13-14)
Supplement: Additional file 1 — Methodological criteria of epidemiological studies. [file 1471-2431-13-14-S1.doc]

| **Additional file 1**  Methodological criteria of epidemiological studies |
| --- |
| **A. Is the final sample representative of the target population?** |
| 1. At least one of the following must apply in the study: an entire target population, randomly selected sample, or sample stated to represent the target population. |
| 2. At least one of the following: reasons for nonresponse described, nonresponders described, comparison of responders and nonresponders, or comparison of sample and target population. |
| 3. Response rate and, if applicable, drop-out rate reported. |
| **B. Quality of the data** |
| 4. Were the data primary data on low back pain or were they taken from a survey not specifically designed for that purpose? |
| 5. Were the data collected from each child or adolescent directly or were they collected from a proxy? |
| 6. Was the same mode of data collection used for all subjects? |
| 7. At least one of the following in case of questionnaire: a validated questionnaire or at least tested for reproducibility. |
| 8. At least one of the following in the case of an interview: Interview validated, tested for reproducibility, or adequately described and standardized. |
| 9. At least one of the following in the case of an examination: Examination validated, tested for reproducibility, or adequately described and standardized. |
| **C. Definition of low back pain (LBP)** |
| 10. Was there a precise anatomic delineation of the lumbar area or reference to an easily obtainable article that contains such specification? |
| 11. Was there further useful specification of the definition of LBP, or question(s) put to study subjects quoted such as the frequency, duration or intensity, and character of the pain. Or was there reference to an easily obtainable article that contains such specification? |
| 12. Were recall periods clearly stated: e.g., 1 week, 1 month or lifetime? |
|  |
