# Supplementary material for: Prevalence of low back pain in children and adolescents: a meta-analysis
Source: BMC Pediatr. 2013 Jan 26;13:14. doi: 10.1186/1471-2431-13-14 (PMC3571904; doi:10.1186/1471-2431-13-14)
Supplement: Additional file 3 — Characteristics of the studies presenting LBP prevalence data used in this meta-analysis. [file 1471-2431-13-14-S3.doc]

| **Additional file 3**  Characteristics of the studies presenting LBP prevalence data used in this meta-analysis | | | | | | | | | | | | |
| --- | --- | --- | --- | --- | --- | --- | --- | --- | --- | --- | --- | --- |
| Study | Year | Continent | Country | Population type | Sample | Age  mean (years) | Males  (%) | Collection mode | Final  sample  size | Response  rate  (%) | Design | Recall periods |
| Auvinen et al. [24]^a^ | 2008 | Europe | Finland | School | A | 15,50 | 47.14 | QM | 5999 | 65 | Cross-sectional | 6M |
| Balagué et al. [11] | 1988 | Europe | Switzerland | School | C + A | 12 | 49 | QI | 1302 | 75,92 | Cross-sectional | L,L* |
| Balagué et al. [44] | 1993 | Europe | Switzerland | School | C + A | 13 | 54 | QI+ I | 113 | 96,6 | Cross-sectional | 1W,L |
| Balagué et al. [45] | 1994 | Europe | Switzerland | School | C + A | 11.70 | 49.40 | QI | 1716 | 97,80 | Cross-sectional | L |
| Balagué et al. [46] | 1995 | Europe | Switzerland | School | A | 14 | 47.50 | QI | 615 | 97,93 | Cross-sectional | 1M*,L |
| Balagué et al. [47] | 2010 | Europe | Switzerland | School + athletes | A | 14 | 100 | I + E | 95 | 100 | Longitudinal | L* |
| Bejia et al. [10] | 2005 | Africa | Tunisia | School | C + A | 14.10 | 47.59 | QI | 622 | 98 | Cross-sectional | 1W,L |
| Burton et al. [4] | 1996 | Europe | UK | School | C | 11.70 | 50 | I | 216 | - | Longitudinal | P,L |
| Çakmak et al. [8] | 2004 | Europe | Turkey | University | A | 17.50 | 47.90 | QI | 1527 | 98,39 | Cross-sectional | L |
| Carvalho [48] | 2009 | Europe | Portugal | School | C + A | 12.70 | 51.13 | QI | 532 | - | Cross-sectional | 1M,1Y |
| Coelho et al. [25] | 2005 | Europe | Portugal | Community | C + A | 12.80 | 49.52 | QI | 208 | - | Cross-sectional | P,1Y,L |
| Diepenmaat et al. [49] | 2006 | Europe | Netherlands | School | A | 14 | 49.53 | QM | 3485 | 77,19 | Cross-sectional | 1M |
| Ebrall [50] | 1994 | Oceania | Australia | School | A | 15.50 | 100 | QI | 610 | 100 | Cross-sectional | P,L |
| El-Metwally et al. [51] | 2008 | Europe | Finland | Community | C | 11,40 | - | QM | 3580 | 71,97 | Cross-sectional | 3M |
| Fairbank et al. [52] | 1984 | Europe | UK | School | A | 15 | 50.90 | QI+ E | 446 | 100 | Cross-sectional | L |
| Feldman et al. [53]^b^ | 1999 | America | Canada | School | A | 13.80 | 52.60 | Q | 502 | 61,98 | Longitudinal | 6M |
| Grimmer y Williams [54] | 2000 | Oceania | Australia | School | A | 14.59 | 56 | QI | 1193 | 57,49 | Cross-sectional | 2W |
| Gunzburg et al. [55]^c^ | 1999 | Europe | Belgium | School | N | 9 | 51.53 | QI+ E | 392 | 100 | Cross-sectional | L |
| Hakala et al. [23]^d^ | 2006 | Europe | Finland | Community | A | 14 | 46.64 | QM | 2337 | 71,8 | Longitudinal | 6M |
| Hangai et al. [56] | 2010 | Asia | Japan | University | A | 18,40 | 56.14 | QI | 4667 | 67,4 | Cross-sectional | 1M,L |
| Harreby et al. [12] | 1999 | Europe | Denmark | School | A | 14.50 | 48.31 | QM | 1389 | 100 | Cross-sectional | P,1W,1M,1Y,L |
| Hestbaek et al. [57]^e^ | 2004 | Europe | Denmark | Community | A | 15.50 | 49.35 | QM | 6693 | 84 | Cross-sectional | P,1Y,L |
| Jones et al. [19] a | 2004 | Europe | UK | School | C + A | 13.50 | 49,80 | Q | 500 | 93 | Cross-sectional | 1W,L |
| Jones et al. [58] b | 2004 | Europe | UK | School | A | 13.90 | 46.10 | QI | 1293 | 97,51 | Cross-sectional | 1M |
| Kaspiris et al. [59] | 2010 | Europe | Greece | Clinical | C + A | 11.20 | 49 | QI + E | 692 | - | Cross-sectional | 1Y |
| Kjaer et al. [60]^f^ | 2005 | Europe | Denmark | School | A | 13.10 | 47 | I | 439 | 74,53 | Cross-sectional | 1M |
| Korovessis et al. [61] | 2004 | Europe | Greece | School | C + A | 12 | 47 | I + E | 3441 | 81,4 | Cross-sectional | P* |
| Kovacs et al. [62] | 2003 | Europe | Spain | School | A | 14 | 47 | QI | 7048 | 97,75 | Cross-sectional | 1W,L |
| Kristensen and Ommundsen [63] | 2001 | Europe | Norway | School | A | 15 | - | QI | 190 | 60 | Cross-sectional | 1Y*,L |
| Kujala et al. [64] | 1992 | Europe | Finland | School + athletes | C + A | 11.80 | 42.03 | QM | 138 | 86,80 | Cross-sectional | 1Y*,L |
| Kujala et al. [65] | 1996 | Europe | Finland | School + athletes | C | 11.80 | 51.02 | QM | 98 | 84,48 | Longitudinal | 1Y* |
| Kujala et al. [66] | 1999 | Europe | Finland | School | C + A | 12.50 | 50.72 | QI | 698 | 100 | Cross-sectional | 1Y* |
| Martínez-Crespo et al. [67] | 2009 | Europe | Spain | School | A | 13.66 | 49.10 | Q | 849 | 95,72 | Cross-sectional | 1Y |
| Masiero et al. [68] | 2008 | Europe | Italy | Community | A | 15 | 50.08 | QI | 7542 |  | Cross-sectional | 1Y* |
| Mierau et al. [69] | 1989 | America | Canada | School | C + A | 11.75 | 48.62 | I | 402 | - | Cross-sectional | 6M |
| Mikkonen et al. [70] | 2008 | Europe | Finland | School | A | 16 | 43.70 | QM | 1987 | 66,92 | Longitudinal | 6M |
| Mohseni-Bandpei et al. [26] | 2007 | Asia | Iran | School | C + A | 13,10 | 47.70 | Q+ E | 4813 | 96,2 | Cross-sectional | P,1M, 6M,1Y |
| Murphy et al. [71]^g^ | 2007 | Europe | UK | School | C + A | 12,80 | 50.50 | QI | 679 | 97 | Cross-sectional | 1M,L |
| Newcomer and Sinaki [72] | 1996 | America | EEUU | School | C + A | 14.30 | 55.21 | QI | 96 | 82,76 | Cross-sectional | 1Y,L |
| Oliveira [73] | 2010 | Europe | Portugal | School | C + A | 12.97 | 45.60 | QI | 239 |  | Longitudinal | 6M |
| Olsen et al. [74] | 1992 | America | EEUU | School | C + A | 13.60 | 51.60 | QI | 1242 | 99,68 | Cross-sectional | 1Y, L |
| Pellise et al. [75] | 2009 | Europe | Spain/ Switzerland | School | A | 15.05 | 52.61 | QI | 1470 | 85,1 | Cross-sectional | 1M |
| Prendeville and Dockrell [76] | 1998 | Europe | Ireland | School | A | 15 | - | QI | 188 | 94 | Cross-sectional | P,1Y,L |
| Prista et al. [77] | 2004 | Africa | Mozambique | School | C + A | 13.50 | 46 | QI | 204 | 85 | Cross-sectional | 1M*,1Y*,L |
| Ratliffe [78] | 2010 | America | EEUU | School | C | 10 | - | QI | 571 | - | Cross-sectional | L |
| Salminen et al. [79] | 1992 | Europe | Finland | School | A | 14 | 51.76 | QI+ E | 1377 | 91,61 | Cross-sectional | 1M,1Y*,L |
| Sato et al. [43]^h^ | 2008 | Asia | Japan | Community | C + A | 12 | 51.24 | QM | 34423 | 78,9 | Cross-sectional | P,L |
| Shebad et al. [20] | 2004 | Asia | Kuwait | School | C + A | 14 | 49.75 | QI + I | 400 | 100 | Cross-sectional | P, 1Y*,L |
| Sjolie and Ljunggren [22]^i^ | 2001 | Europe | Norway | School | A | 14.70 | 56.82 | QI | 88 | 83,81 | Cross-sectional | 1Y,L |
| Skoffer [9]^j^ | 2007 | Europe | Denmark | School | A | 15,50 | 53,30 | QI | 546 | 87,7 | Cross-sectional | 3M,3M*,1Y,L |
| Staes et al. [80] | 2003 | Europe | Belgium | School | A | 17.08 | 46.93 | QI | 620 | 84,82 | Cross-sectional | 1M |
| Taimela et al. [14] | 1997 | Europe | Finland | School | C + A | 11.50 | 47.27 | QI | 1171 | 82 | Cross-sectional | 1Y* |
| Trevelyan and Legg [81] | 2010 | Oceania | New Zealand | School | C + A | 12.02 | 45,71 | QI | 245 | 18,41 | Cross-sectional | 1M |
| Troussier et al. [82] | 1994 | Europe | France | School | C + A | 12.80 | 46.90 | QI | 1176 | 90,70 | Cross-sectional | L |
| Vikat et al. [83] | 2000 | Europe | Finland | Community | A | 15 | - | QM | 11095 | 75,40 | Cross-sectional | 6M |
| Watson et al. [84]^k^ | 2002 | Europe | UK | School | C + A | 12.50 | 46.10 | QI | 1376 | 91,98 | Cross-sectional | 1M |
| Wedderkopp et al. [85]^l^ | 2001 | Europe | Denmark | School | C + A | 12.14 | 48 | I | 806 | 59,44 | Cross-sectional | 1M |
| Whittfield et al. [21] | 2005 | Oceania | New Zealand | School | A | 15.35 | 50 | QI | 140 | 100 | Cross-sectional | 1W |
| Young et al. [86] | 2006 | America | EEUU | School | C + A | 11.20 | 41.50 | QM | 184 | 27,5 | Cross-sectional | P |
| C: Children; A: Adolescent; P: Point; Q: Questionnaire; QI: Questionnaire “in situ”; QM: Questionnaire massive; I: Interview; E: Examination; W: Week; M: Month; Y: Year; L: Lifetime; *Disability; ^a^Data collected from [87,88]; ^b^Data collected from [12]; ^c^Data collected from [89]; ^d^Data collected from [90]; ^e^Data collected from [91,92]; ^f^Data collected from [93]; ^g^Data collected from [94]; ^h^Data collected from [95]; ^i^Data collected from [6,96-98]; ^j^Data collected from [99]; ^k^Data collected from [100,101]; ^l^Data collected from [102,103]. | | | | | | | | | | | | |
