# Supplementary material for: Prevalence of low back pain in children and adolescents: a meta-analysis
Source: BMC Pediatr. 2013 Jan 26;13:14. doi: 10.1186/1471-2431-13-14 (PMC3571904; doi:10.1186/1471-2431-13-14)
Supplement: Additional file 4 — Methodological Quality of the 59 epidemiological studies. [file 1471-2431-13-14-S4.doc]

| **Additional file 4**  Methodological Quality of the 59 epidemiological studies | | | | | | | | | | | | | |
| --- | --- | --- | --- | --- | --- | --- | --- | --- | --- | --- | --- | --- | --- |
| Criterion Study | 1 | 2 | 3 | 4 | 5 | 6 | 7 | 8 | 9 | 10 | 11 | 12 | Total  score  % |
| Auvinen et al. [24] 2008 | + | + | + | + | - | + | + | NA | NA | + | - | + | 80 |
| Balagué et al. [11] 1988 | + | - | + | + | + | + | + | NA | NA | - | - | + | 70 |
| Balagué et al. [44] 1993 | + | - | + | + | + | + | + | + | NA | - | - | + | 73 |
| Balagué et al. [45] 1994 | + | - | + | + | + | + | + | NA | NA | - | + | + | 80 |
| Balagué et al. [46] 1995 | + | - | + | + | + | + | + | NA | NA | + | - | + | 80 |
| Balagué et al. [47] 2010 | - | NA | + | + | + | + | NA | + | + | + | + | + | 90 |
| Bejia et al. [10] 2005 | + | + | + | + | + | + | + | NA | NA | + | + | + | 100 |
| Burton et al. [4 1996 | - | - | - | + | + | + | NA | + | NA | - | - | - | 40 |
| Çakmak et al. [8] 2004 | + | - | + | + | + | + | - | NA | NA | + | + | + | 80 |
| Carvalho [48] 2009 | - | - | - | + | + | + | + | NA | NA | + | + | + | 70 |
| Coelho et al. [25] 2005 | - | - | - | + | + | + | + | NA | NA | + | + | + | 70 |
| Diepenmaat et al. [49] 2006 | + | - | + | + | + | + | + | NA | NA | + | + | + | 90 |
| Ebrall [50] 1994 | - | NA | + | + | + | + | + | NA | NA | + | + | + | 89 |
| El-Metwally et al. [51] 2008 | + | - | + | + | - | + | + | NA | NA | + | + | + | 80 |
| Fairbank et al. [52] 1984 | - | NA | + | + | + | + | + | NA | + | - | + | + | 80 |
| Feldman et al. [53] 1999 | + | - | + | + | - | + | - | NA | NA | - | - | + | 50 |
| Grimmer y Williams [54] 2000 | + | + | + | + | + | + | + | NA | NA | - | - | + | 80 |
| Gunzburg et al. [55] 1999 | + | NA | + | + | + | + | + | NA | + | + | - | + | 90 |
| Hakala et al. [23] 2006 | + | - | + | + | - | + | + | NA | NA | - | + | + | 70 |
| Hangai et al. [56] 2010 | + | + | + | + | + | + | + | NA | NA | + | - | + | 90 |
| Harreby et al. [12] 1999 | + | NA | + | + | + | + | + | NA | NA | + | + | + | 100 |
| Hestbaek et al. [57] 2004 | + | - | + | - | + | + | + | NA | NA | + | - | + | 70 |
| Jones et al. [19] 2004 a | - | - | + | + | - | + | + | NA | NA | + | + | + | 70 |
| Jones et al. [58] 2004 b | + | - | + | + | + | + | + | NA | NA | - | - | + | 70 |
| Kaspiris et al. [59] 2010 | + | - | - | + | + | + | + | NA | + | + | + | + | 82 |
| Kjaer et al. [60] 2005 | + | - | + | + | + | + | NA | + | NA | - | - | + | 70 |
| Korovessis et al. [61] 2004 | + | + | + | + | + | + | NA | - | + | + | - | + | 82 |
| Kovacs et al. [62] 2003 | + | + | + | + | + | + | + | NA | NA | - | + | + | 90 |
| Kristensen and Ommundsen [63] 2001 | + | - | + | + | + | + | + | NA | NA | + | - | + | 80 |
| Kujala et al. [64] 1992 | - | - | + | + | - | + | + | NA | NA | + | - | + | 60 |
| Kujala et al. [65] 1996 | - | - | + | + | - | + | + | NA | NA | + | - | - | 50 |
| Kujala et al. [66] 1999 | + | NA | + | + | + | + | + | NA | NA | + | - | + | 89 |
| Martínez-Crespo et al. [67] 2009 | + | - | + | + | + | + | + | NA | NA | - | + | + | 80 |
| Masiero et al. [68] 2008 | + | - | - | + | + | + | - | NA | NA | + | + | + | 70 |
| Mierau et al. [69] 1989 | - | - | - | + | + | + | NA | - | NA | - | - | + | 40 |
| Mikkonen et al. [70] 2008 | + | + | + | + | - | - | + | NA | NA | + | - | + | 70 |
| Mohseni-Bandpei et al. [26] 2007 | + | - | + | + | + | + | + | NA | + | + | - | + | 82 |
| Murphy et al. [71] 2007 | + | - | + | + | + | + | + | NA | NA | + | - | + | 80 |
| Newcomer and Sinaki [72] 1996 | - | - | + | + | + | + | + | NA | NA | - | - | + | 60 |
| Oliveira [73] 2010 | - | - | - | + | + | + | + | NA | + | + | + | + | 73 |
| Olsen et al. [74] 1992 | + | - | + | + | - | + | + | NA | NA | - | - | + | 60 |
| Pellise et al. [75] 2009 | + | + | + | + | + | + | + | NA | NA | + | + | + | 100 |
| Prendeville and Dockrell [76] 1998 | + | - | + | + | + | + | + | NA | NA | + | + | + | 90 |
| Prista et al. [77] 2004 | + | + | + | + | + | + | + | NA | NA | - | - | + | 80 |
| Ratliffe [78] 2010 | - | - | - | + | + | + | + | NA | NA | + | - | + | 60 |
| Salminen et al. [79] 1992 | + | - | + | + | + | + | + | NA | - | + | + | + | 82 |
| Sato et al. [43] 2008 | + | - | + | + | + | + | + | NA | NA | - | + | + | 80 |
| Shebad et al. [20] 2004 | + | NA | + | + | + | + | - | - | NA | + | + | + | 80 |
| Sjolie and Ljunggren [22] 2001 | - | - | + | + | + | + | + | NA | NA | + | + | + | 80 |
| Skoffer [9] 2007 | + | + | + | + | + | + | - | NA | NA | + | + | + | 90 |
| Staes et al. [80] 2003 | + | + | + | + | + | + | + | NA | NA | + | + | + | 100 |
| Taimela et al. [14] 1997 | + | + | + | + | + | + | + | NA | NA | - | + | + | 90 |
| Trevelyan and Legg [81] 2010 | + | - | + | + | + | + | + | NA | NA | + | + | + | 90 |
| Troussier et al. [82] 1994 | + | - | + | + | + | + | + | NA | NA | - | + | + | 80 |
| Vikat et al. [83] 2000 | + | - | + | - | - | + | + | NA | NA | - | + | + | 60 |
| Watson et al. [84] 2002 | + | + | + | + | + | + | + | NA | NA | + | + | + | 100 |
| Wedderkopp et al. [85] 2001 | + | + | + | + | + | + | NA | + | NA | - | - | + | 80 |
| Whittfield et al. [21] 2005 | + | NA | + | - | + | + | + | NA | NA | - | - | + | 67 |
| Young et al. [86] 2006 | - | - | + | + | - | + | - | NA | NA | - | - | + | 40 |
| + = criterion fulfilled; - = criterion not fulfilled; NA = not applicable.  Representativeness = columns 1 to 3.  Quality of Date = columns 4 to 9.  Definition of low back pain = columns 10 to 12. | | | | | | | | | | | | | |
